# Effect of Cyclosporine on Lesion Growth and Infarct Size within the White and Gray Matter

**DOI:** 10.3389/fneur.2017.00151

**Published:** 2017-04-27

**Authors:** Elodie Ong, Nathan Mewton, Julien Bouvier, Fabien Chauveau, Thomas Ritzenthaler, Laura Mechtouff, Laurent Derex, Marielle Buisson, Yves Berthezène, Michel Ovize, Norbert Nighoghossian, Tae-Hee Cho

**Affiliations:** ^1^Department of Stroke Medicine, Université Lyon 1, Lyon, France; ^2^Department of Neuroradiology, Université Lyon 1, Lyon, France; ^3^CREATIS, CNRS-UMR5220 INSERM-U1044, Lyon, France; ^4^INSA-Lyon, Lyon, France; ^5^Hospices Civils de Lyon, Lyon, France; ^6^Department of Cardiology, Clinical Investigation Center, Université Lyon 1, Lyon, France; ^7^CarMeN, CNRS-UMR1060, Lyon, France; ^8^Hospices Civils de Lyon, Lyon, France; ^9^Lyon Neuroscience Research Center, Université Lyon 1, Lyon, France; ^10^CNRS-UMR5292, Lyon, France; ^11^INSERM-U1028, Lyon, France

**Keywords:** ischemic stroke, thrombolysis, neuroprotection, recanalization, cyclosporin

## Abstract

**Background:**

In a recent trial, cyclosporine A (CsA) failed to reduce infarct size in acute stroke patients treated with intravenous thrombolysis. White matter (WM) and gray matter (GM) may have distinct vulnerability to ischemia and response to therapy. Using final infarct size and lesion growth as endpoints, our objectives were to (1) investigate any tissue-specific effect of CsA and (2) compare WM and GM response to thrombolysis.

**Materials and methods:**

We analyzed 84 patients from the randomized and placebo-controlled CsA-Stroke trial, who underwent MRI both on admission and at 1 month. Lesion growth was defined voxel-wise as infarcted tissue at 1 month with no visible lesion on baseline diffusion-weighted imaging. After automatic segmentation of GM/WM, final infarct size and lesion growth were compared within the GM and WM.

**Results:**

Occlusion level was distal (>M1) in 51% of cases. No significant difference in GM/WM proportions was observed within final infarcts between treatment groups (*P* = 0.21). Infarct size within the GM or WM was similar between the CsA and control groups [GM: 9.2 (2.4; 22.8) with CsA vs 8.9 (3.7; 28.4) mL with placebo, *P* = 0.74; WM: 9.9 (4.7; 25.4) with CsA vs 14.1 (5.6; 34.1) mL with placebo, *P* = 0.26]. There was no significant effect of CsA on lesion growth in either the GM or WM. Pooling all patients, a trend for increased relative lesion growth in WM compared to GM was observed [49.0% (14.7; 185.7) vs 43.1% (15.4; 117.1), respectively; *P* = 0.12].

**Conclusion:**

No differential effect of CsA was observed between WM and GM. Pooling all patients, a trend toward greater lesion growth in WM was observed.

## Introduction

Reperfusion can induce additional injury following prolonged periods of ischemia. CsA may limit reperfusion injury by inhibiting several mechanistic pathways: mitochondrial permeability transition pore opening, oxidative stress, microglial activation, and apoptosis (1). CsA has reduced infarct size after percutaneous coronary intervention in acute myocardial infarction (2). However, in a recent phase 2 trial, CsA failed to significantly reduce infarct size in acute stroke patients treated with intravenous tissue plasminogen activator (tPA) (3).

The failure of neuroprotection trials may in part stem from insufficient distinction between white matter (WM) and gray matter (GM) (4). Indeed, few studies compared the vulnerability of WM and GM during ischemia, or their respective response to therapy (5–7). The effects of CsA may differ between these two compartments, which exhibit distinct cellular structure, metabolic, and hemodynamic requirements.

In this study involving patients treated with intravenous thrombolysis (no patient underwent mechanical thrombectomy), our objectives were to compare the final infarct size and lesion growth within WM and GM in (1) CsA vs placebo-treated patients, so as to assess any tissue-specific effect of CsA, and (2) a pooled analysis of all patients, to compare WM and GM response to ischemia and thrombolysis.

## Patients and Methods

### Patients

Cyclosporine A-stroke was a multicenter, randomized, single-blinded phase-II trial that enrolled patients aged 18–85 years presenting with an anterior-circulation stroke, a National Institutes of Health Stroke Scale score between 6 and 18, and who were treated by intravenous tPA within 4.5 h of symptoms onset. Patients received either a single intravenous bolus of CsA (2.0 mg/kg, Sandimmune, Novartis) or placebo. The primary endpoint was infarct size mapped on MRI at 1 month (3). For the present study, only patients with complete and assessable MRI at baseline and 1 month were included.

### MRI Protocol

MRI was the first-line imaging method on admission and included diffusion-weighted imaging (DWI), T2*, fluid-attenuated inversion-recovery (FLAIR) and time-of-flight angiography. The same protocol was repeated at 1 month.

### Image Analysis

**Figure 1:**
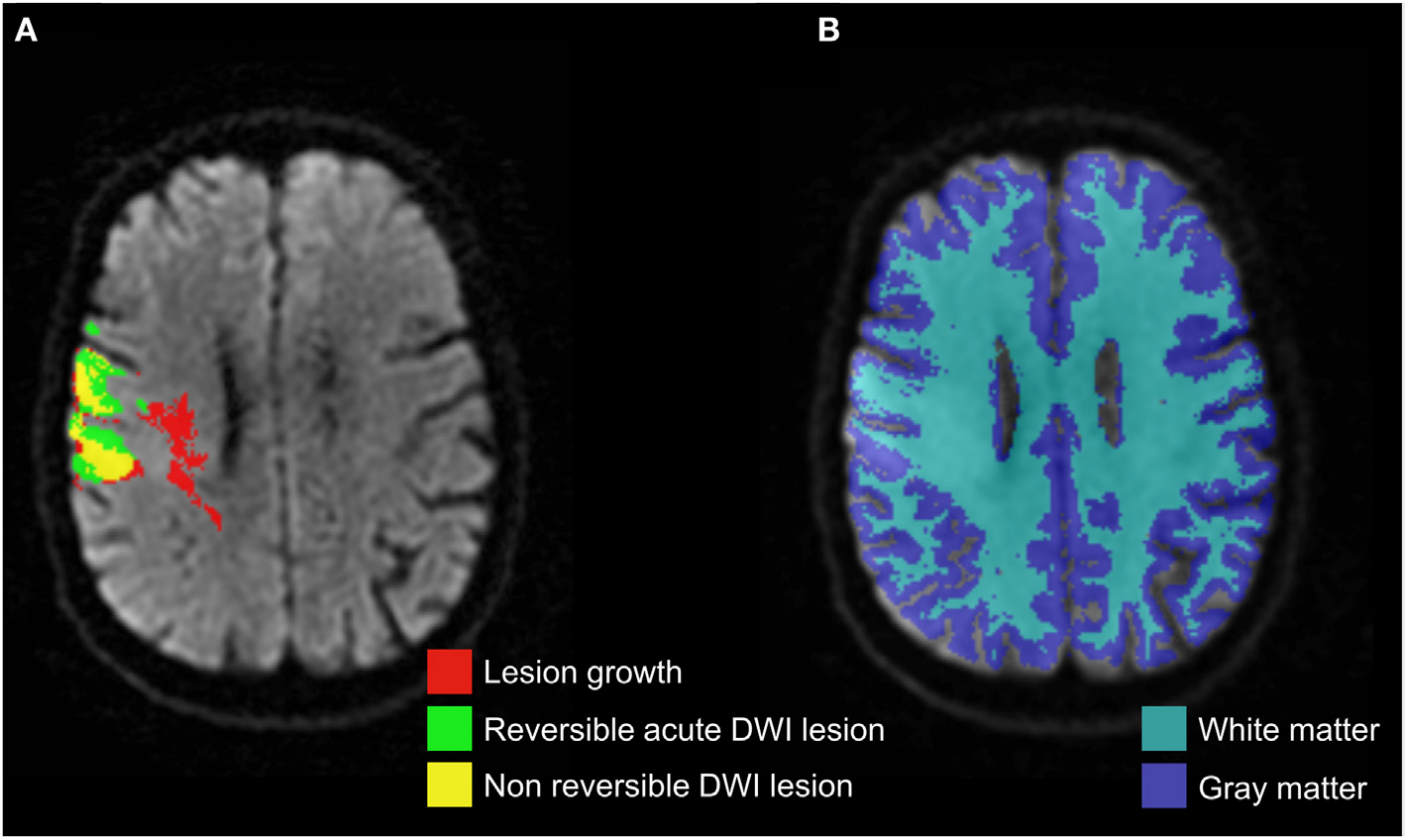
**Co-registered masks of acute diffusion-weighted imaging (DWI) lesion, final infarct, and corresponding lesion growth (A) overlaid on acute DWI, which were then segmented by the gray matter and white matter probabilistic maps (B)**.

Baseline DWI lesions and final infarcts on FLAIR were outlined as previously described (3). WM and GM were individually segmented from baseline T2* images using SPM8 (London, UK) (6). All baseline and 1-month images were co-registered within subjects. Lesion growth was defined as voxels included in the final FLAIR lesion, but not in the baseline DWI lesion. WM and GM masks were used to assess the volume and proportion of WM and GM within the final infarct and lesion growth (Figure 1). Image analyses were performed using Matlab (MathWorks, USA).

### Statistical Analyses

Results were described as proportions or median with interquartile range. Comparisons between treatment groups were performed with the Student’s *t*-test or Wilcoxon ranked-sum test, and with the Chi-square or Fisher’s exact test, as appropriate. A repeated-measures ANOVA compared the final infarct size and lesion growth, globally and within GM and WM. Analyses were performed using STATA 13.0 (StataCorp, USA). *P*-values <0.05 were considered significant.

## Results

From October 2009 to July 2013, 127 patients were enrolled in CsA-Stroke. Forty-three patients were excluded from the present study because of incomplete/missing baseline MRI or images of insufficient quality. Thus, 84 patients were analyzed: 43 and 41 in the control and CsA group, respectively. Baseline characteristics are summarized in the Table 1. Overall, occlusion level was distal (>M1) in 43 patients (51%).

**Table 1:** **Patients’ baseline characteristics**.

Parameter	CsA	Control	*P*-value
Age	65.6 ± 11.4	67.8 ± 13.5	0.18
Male, *n* (%)	23 (56)	19 (44)	0.38
NIHSS, mean (min–max)	12 (4–18)	13 (6–19)	0.24
Time from onset to MRI, min	121 ± 47	111 ± 44	0.09
Delay to thrombolysis, min	161 ± 47	149 ± 46	0.09
DWI lesion (mL), median (IQR)	18.9 (6.7; 44.5)	14.7 (8.0; 39.9)	0.71
Occlusion level, distal/proximal	21/20	22/21	1.0
Recanalization, *n* (%)	29 (70)	25 (58)	0.12

*Results are presented as mean ± SD unless specified otherwise*.

*CsA, cyclosporine A; NIHSS, National Institutes of Health Stroke Scale; MRI, magnetic resonance imaging; DWI, diffusion-weighted imaging; IQR, interquartile range*.

### CsA vs Placebo

**Figure 2:**
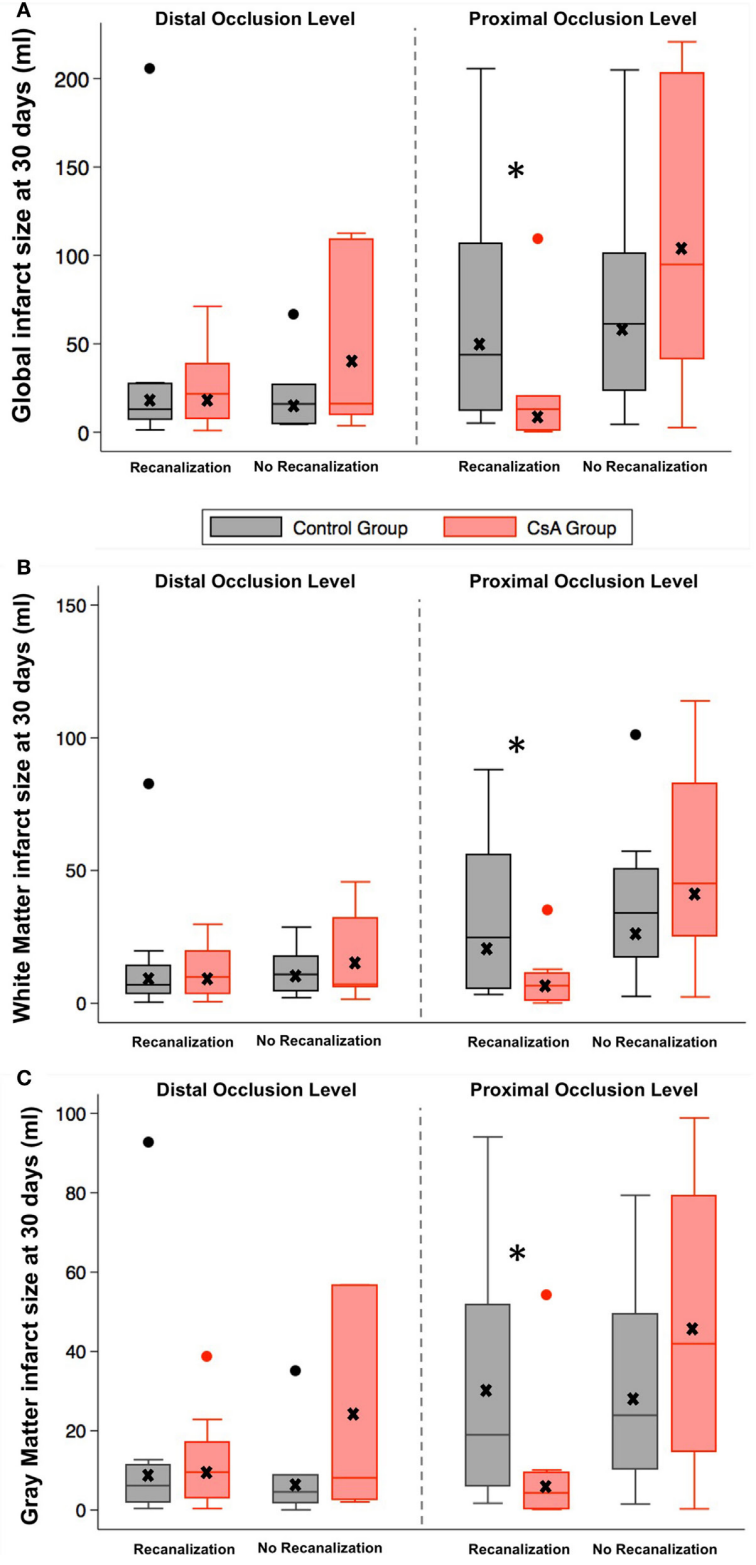
**Infarct size at 1 month according to recanalization status, occlusion level, and treatment groups in the whole brain (A), white matter (B), and gray matter (C)**. Within boxplots: horizontal line indicates the median; lower and upper limits: first and third quartiles; whiskers are placed at 1.5 times the interquartile range; crosses indicate the mean, *P = 0.02.

Cyclosporine A did not significantly reduce infarct size at 1 month compared to placebo [20.6 mL (6.8; 53.8) vs 26.9 mL (11.3; 66.3), respectively; *P* = 0.38], nor lesion growth [9.2 mL (2.1; 19.8) vs 10.1 mL (2.7; 33.9), respectively; *P* = 0.38]. As in the main study, CsA-treated patients with a proximal occlusion and subsequent recanalization had smaller infarct volumes compared to controls [13.1 mL (1.2; 20.3) vs 43.8 mL (12.3; 106.7), respectively; *P* = 0.02; Figure 2A], as well as reduced lesion growth [3.0 mL (0.3; 9.4) vs 17.1 mL (5.2; 70.9), respectively; *P* = 0.01]. No significant difference in GM/WM proportions was observed within final infarcts between treatment groups [GM/WM (mean ± SD): 46.4 ± 12.6/53.6 ± 12.6% for CsA vs 42.6 ± 15.3/57.4 ± 15.3% for placebo; *P* = 0.21]. Infarct size within the GM or WM was similar between the CsA and control groups [GM: 9.2 mL (2.4; 22.8) with CsA vs 8.9 mL (3.7; 28.4) with placebo, *P* = 0.74; WM: 9.9 mL (4.7; 25.4) with CsA vs 14.1 mL (5.6; 34.1) with placebo, *P* = 0.26]. Again, CsA significantly reduced infarct size in patients with proximal occlusion and recanalization, in both the GM [4.3 mL (0.3; 9.4) with CsA vs 19.0 mL (6.1; 51.8) in controls; *P* = 0.048] and WM [6.6 mL (0.9; 11.1) in CsA group vs 24.8 mL (5.6; 55.8) in controls; *P* = 0.01; Figures 2B,C]. There was no significant effect of CsA on relative lesion growth compared to placebo in either the GM or WM [GM: 33.7% (15.9; 83.4) with CsA vs 60.8% (14.2; 152.9) in controls, *P* = 0.35; WM: 32.8% (14.3; 159.0) with CsA vs 65.6% (15.2; 208.3) in controls, *P* = 0.51].

### WM vs GM in Pooled Analyses

As CsA had overall no impact on imaging endpoints, we pooled all patients (CsA and placebo) to further explore any difference between GM and WM. Absolute infarct size was similar between GM and WM [9.2 mL (2.5; 26.1) vs 11.7 mL (5.5; 29.9), respectively; *P* = 0.20]. However, final infarcts had greater proportions of WM than GM [53.3% (46.9; 64.2) vs 46.7% (35.8; 53.0), respectively; *P* = 0.0005]. Absolute lesion growth was larger in WM compared to GM [5.3 mL (1.4; 13.5) vs 3.9 mL (1.1; 10.7), respectively; *P* = 0.012]. A trend for increased relative lesion growth in WM compared to GM was observed [49.0% (14.7; 185.7) vs 43.1% (15.4; 117.1), respectively; *P* = 0.12].

## Discussion

In this study, we found no differential effect of CsA between WM and GM. Tissue-specific measurements showed no benefit of CsA in reducing lesion growth or infarct volume. CsA administration improved these endpoints in patients with proximal occlusion and subsequent recanalization, but to a similar extent in WM and GM. The potential benefit of CsA in this subgroup will require further evaluation, but our results do not suggest a tissue-specific response.

Whether GM and WM have a distinct vulnerability to ischemia remains controversial. Higher ischemic thresholds are found in GM compared to WM, but this may only reflect the inherent gap in metabolism and hemodynamics in these compartments (8). Preclinical studies using different animal models yielded conflicting results (9, 10). Positron-emission tomography and MRI-based clinical reports showed higher proportions of penumbral tissue within the WM compared to GM, suggesting a greater tolerance to ischemia in the former (5, 6). However, in these untreated patients, similar proportions of at-risk WM and GM eventually infarcted. In our study, there was a trend toward increased lesion growth in WM in a pooled analysis of patients treated with intravenous tPA. This may suggest tissue-specific responses to ischemia-reperfusion, but needs confirmation in an adequately powered study.

Our report has some limitations. Reperfusion, penumbral tissue, and its evolution were not monitored, as perfusion-weighted imaging was not systematically performed. The lack of high-resolution T1-weighted imaging may have impaired the precision of WM/GM segmentation. The main CsA-Stroke study was negative; the present report based on subgroup analyses may have an insufficient sample size and thus be underpowered to assess the differential effects of CsA in the WM and GM.

In conclusion, in our population of patients with distal or proximal occlusions, CsA had no significant effect on lesion growth and final infarct size in either the WM or GM. A trend toward greater lesion growth in WM was observed when pooling all patients.

## Ethics Statement

This study was carried out in accordance with the recommendations of the regional ethical standards committee on human experimentation (Comité de Protection des Personnes Sud Est IV, Centre Léon Bérard, 28 rue Laennec, 69373 Lyon Cedex 08) with written informed consent from all subjects. The national regulatory authority approved the study, and guaranteed that it conformed to the regulatory standard (AFSSAPS No. A90656-22). All subjects gave written informed consent in accordance with the Declaration of Helsinki. The protocol was approved by the Comité de Protection des Personnes Sud Est IV. The trial was registered at http://ClinicalTrials.gov (NCT01527240) and EudraCT (2009-012590-35).

## Author Contributions

EO and T-HC: data acquisition, analysis, manuscript drafting, and critical revision. NW: data analysis, statistical analysis, and critical revision of the manuscript. JB and FC: data analysis and critical revision of the manuscript. TR, LM, LD, and MB: data acquisition and critical revision of the manuscript. YB and NN: study design, data acquisition, analysis, and critical revision of the manuscript. MO: study design, analysis, and critical revision of the manuscript.

## Conflict of Interest Statement

The authors declare that the research was conducted in the absence of any commercial or financial relationships that could be construed as a potential conflict of interest.

## CsA-Stroke Investigators

**Agnès Jacquin**, Department of Neurology, University Hospital of Dijon, Regional Council and University of Burgondy, CHU le Bocage, Dijon, France; **Benoit Guillon**, Department of Neurology, Hôpital Nord-Laënnec, Université de Nantes, Saint-Herblain, France; **Catherine Lamy**, Department of Neurology, CH Sainte-Anne, University Paris-Descartes, INSERM U894, DHU NeuroVasc Sorbonne Paris-Cité, Paris, France; **Catherine Oppenheim**, Department of Neuroradiology, CH Sainte-Anne, University Paris-Descartes, INSERM U894, DHU NeuroVasc Sorbonne Paris-Cité, Paris, France; **Eric Bodiguel**, Department of Neurology, CH Sainte-Anne, University Paris-Descartes, INSERM U894, DHU NeuroVasc Sorbonne Paris-Cité, Paris, France; **Frédéric Philippeau**, Department of Neurology, centre hospitalier de Fleyriat, Bourg en Bresse, France; **Frédéric Ricolfi**, Department of Neuroradiology, University Hospital of Dijon, Regional Council and University of Burgondy, CHU le Bocage, Dijon, France; **Guillaume Turc**, Department of Neurology, CH Sainte-Anne, University Paris-Descartes, INSERM U894, DHU NeuroVasc Sorbonne Paris-Cité, Paris, France; **Hassan Hosseini**, Department of Neurology, Hôpital Henri Mondor, Université Paris-Est Créteil, Créteil, France; **Hubert Desal**, Department of Neurology, Hôpital Nord-Laënnec, Université de Nantes, Saint-Herblain, France; **Igor Sibon**, Department of Neurology, Groupe Hospitalier Pellegrin, Université de Bordeaux, Bordeaux, France; **Jean-Louis Mas**, Department of Neurology, CH Sainte-Anne, University Paris-Descartes, INSERM U894, DHU NeuroVasc Sorbonne Paris-Cité, Paris, France; **Marie-Hélène Mahagne**, Unité Neurosciences, Hôpital Saint-Roch, Université Nice-Sophia Antipolis, Nice, France; **Mathieu Sevin-Allouet**, Department of Neurology, Hôpital Nord-Laënnec, Université de Nantes, Saint-Herblain, France; **Maurice Giroud**, Department of Neurology, University Hospital of Dijon, Regional Council and University of Burgondy, CHU le Bocage, Dijon, France; **Pierre Amarenco**, Department of Neurology, Hôpital Bichat-Claude Bernard, Université Paris Diderot-Paris 7, Paris, France; **Serkan Cakmak**, Department of Neurology, Hôpital Nord-Ouest, Plateau d’Ouilly, Villefranche sur Saône, France; **Sylvain Rheims**, Department of Neurology, Hôpital Neurologique Pierre Wertheimer, Université Lyon 1, Lyon, France; **Valérie Domigo**, Department of Neurology, CH Sainte-Anne, University Paris-Descartes, INSERM U894, DHU NeuroVasc Sorbonne Paris-Cité, Paris, France; **Vincent Guiraud**, Department of Neurology, CH Sainte-Anne, University Paris-Descartes, INSERM U894, DHU NeuroVasc Sorbonne Paris-Cité, Paris, France; **Yannick Béjot**, Department of Neurology, University Hospital of Dijon, Regional Council and University of Burgondy, CHU le Bocage, Dijon, France.
