# Supplementary material for: Effect of Cyclosporine on Lesion Growth and Infarct Size within the White and Gray Matter
Source: Front Neurol. 2017 Apr 27;8:151. doi: 10.3389/fneur.2017.00151 (PMC5406390; doi:10.3389/fneur.2017.00151)
Supplement: Supplementary file 1 [file Data_Sheet_1.docx]

# Effect of cyclosporine on lesion growth and infarct size within the white and gray matter

Elodie Ong, MD;^1^ Nathan Mewton, PhD;^2^ Julien Bouvier, PhD;^1^ Fabien Chauveau, PhD;^3^ Thomas Ritzenthaler, MD;^1^ Laura Mechtouff, MD;^1^ Laurent Derex, PhD;^1^ Marielle Buisson, PharmD;^2^ Yves Berthezène, PhD;^1^ Michel Ovize, PhD;^2^ Norbert Nighoghossian, PhD;^1^ Tae-Hee Cho, PhD;^1^ on behalf of the CsA-Stroke Investigators.

**Supplemental data**

**CSA effect according to recanalization status**

Global Infarct size (WM+GM)

|  | CSA group | Controls | P value |
| --- | --- | --- | --- |
| Recanalization | 21±21 ml | 41±53 ml | 0.07 |
| No recanalization | 77±67 ml | 50±48 ml | 0.20 |

Infarct size White Matter

|  | CSA group | Controls | P value |
| --- | --- | --- | --- |
| Recanalization | 10±9 ml | 21 ±25 ml | 0.051 |
| No recanalization | 39±35 ml | 27±25 ml | 0.28 |

Infarct Size Gray Matter

|  | CSA group | Controls | P value |
| --- | --- | --- | --- |
| Recanalization | 10±12 ml | 20±28 ml | 0.12 |
| No recanalization | 38±33 ml | 23±25 ml | 0.16 |
